# A Hepatocellular Carcinoma Case in a Patient Who had Immunity to Hepatitis B Virus Earlier

**DOI:** 10.5005/jp-journals-10018-1173

**Published:** 2016-07-09

**Authors:** Ihsan Ates, Mustafa Kaplan, Selim Demirci, Emin Altiparmak

**Affiliations:** 1 Department of Internal Medicine, Ankara Numune Education and Research Hospital, Sihhiye, Ankara, Turkey; 2 Department of Gastroenterology, Ankara Numune Education and Research Hospital, Sihhiye, Ankara, Turkey

**Keywords:** Hepatitis B virus, Hepatitis B e antigen, Hepatitis B surface antigen.

## Abstract

Hepatocellular carcinoma (HCC) is the most common malignant tumor of the liver. Hepatitis B virus infection is one of the most important etilogical factors of HCC. In this case report, a patient with HCC previously infected and having ongoing immunity against hepatitis B virus will be discussed.

**How to cite this article:**

Ates I, Kaplan M, Demirci S, Altiparmak E. A Hepatocellular Carcinoma Case in a Patient Who had Immunity to Hepatitis B Virus Earlier. Euroasian J Hepato-Gastroenterol 2016;6(1):82-83.

## INRODUCTION

Hepatocellular carcinoma (HCC) is the most common primary malignant tumor of the liver.^1,2^ Major risk factors for the development of HCC have been identified.^3^ The relationship between hepatitis B virus (HBV) and HCC has been shown by numerous studies.^4-7^ Hepatocellular carcinoma usualy occurs in patients with chronic hepatitis B with liver cirrhosis. Indeed, HCC may also develop in HBV-infected non-cirrhotic patients. In HBV-infected individuals, additional risk factors for HCC development are viral load and the presence of hepatitis B e antigen and hepatitis B surface antigen.^8-10^ Although HCC is seen in patients with active viral replication, it can also be seen in inactive HBV carriers, occult HBV-infected patients, and immunocompromised patients.^7,11,12^ In this case report, the devolopment of HCC in a patient with past HBV infection and with natural immunity against HBV will be discussed.

## CASE REPORT

A 53-year-old male patient with pain in the right hypochondriac region attended for diagnosis and management. In the upper abdominal ultrasonography, a lession that filled all of the right lobe and a portion of the left lobe of the liver was identified and the patient was referred to the gastroenterology clinic. Laboratory findings are as follows: Alanine aminotransferase: 55 U/L, aspartate aminotransferase: 58 U/L, gamma glutamyl transferase: 639 U/L, alkaline phosphatase: 400 U/L, and alpha fetoprotein: 1210 ng/mL. Hepatobiliary ultrasonography revealed a lesion of about 12 cm in diameter, filling the right lobe of the liver, and heterogeneous in characeter. Upper abdomen magnetic resorance imaging (MRI) showed a lession that covered the anterior and posterior segments of the right lobe of the liver and had heterogeneous-hypointense components in T1-weighted sequences and heterogeneous hypo- or hyperintense components in T2-weighted sequences. After IVCM injection, the heterogeneous pattern of enhancement mass was observed. In all stages of dynamic images, a heterogeneous pattern of contrast enhancement was seen. Around the lession, multiple satellite lesions were monitored. After that, needle biopsy of the lesion was performed by interventional radiology. Patholgical assessment revelaed a solid type of HCC. There was no history of chronic diseases, drug use, smoking, and alcohol use. There was no family history of malignancy. The upper abdomen ultrasonography that was accomplsihed 1 year earlier did not show any lesion or hepatic-steatosis. To investigate the etiology of HCC iron, iron-binding capacity, ferritin, 24-hour urine copper, ceruloplasmin, alpha-1 antitrypsin level, antinuclear antibody, antimitochondrial antibody, antismooth muscle antibody, soluble liver antigen, and liver kidney microsomal enzyme levels were measured and all these parameters were within normal limits. In serological tests, anti-HBs was 29.84 S/CO (positive), whereas the patient was anti-HBe and HBeAg negative. The total anti-HBc was positive (2.62 S/CO); however, anti-HBc IgM (0.05 S/CO), HBs Ag (0.26 S/CO), anti-HCV antiobody (0.11 S/CO), and anti-HIV (0.1 S/CO) were negative. Also, HBV DNA, HCV RNA, and DELTA antibody could not be detected in this patient.

## DISCUSSION

Cirrhosis is a major risk factor for the development of HCC. The main factors for the development of cirrhosis are HBV and HCV infection. Worldwide, 50% of HCC cases are due to HBV infection and 25% are due to HCV infection.^13^ Approximately 15% of HCC cases develop in the non-cirrhotic liver.^14^ Our patient did not have cirrhosis. The risk factors for the devolopment of HCC in patients with chronic HBV infection are viral load and the presence of HBeAg and HbsAg.^8-10^ A study conducted in Taiwan between 1991 and 1992 revealed that the risk of devolopment of HCC increased as HBV DNA copy number increased in HBsAg-positive and anti-HCV-negative patients.^8^ Another prospective study was conducted in Taiwan on 11,893 men who were suffering from chronic hepatitis B. After 10 years of follow-up, HCC developed in 111 patients. The incidence of HCC in both HBeAg- and HBsAg positive patients was higher than in patients who were only HBsAg positive or both negative.^15^ Also, it was shown that inactive HBV carriers, posttreatment HBV-infected patients, and patients with natural immunity to HBV (anti-HBc total: positive, anti-HBs positive) had higher HCC devolopment risk than the normal population sharing the same demographic features.^7^ This indicates that HCC can develop in patients with natural immunity against HBV.^12,14^ In our case, HBsAg was found negative; however, anti-HBc and anti-HBs were positive.
